# Emergence of swine influenza A virus, porcine respirovirus 1 and swine orthopneumovirus in porcine respiratory disease in Germany

**DOI:** 10.1080/22221751.2023.2239938

**Published:** 2023-08-02

**Authors:** Annika Graaf-Rau, Christin Hennig, Kathrin Lillie-Jaschniski, Monika Koechling, Julia Stadler, Jan Boehmer, Ulrike Ripp, Anne Pohlmann, Bernd-Andreas Schwarz, Martin Beer, Timm Harder

**Affiliations:** a Institute of Diagnostic Virology, Friedrich-Loeffler-Institut, Greifswald-Insel Riems, Germany; b Ceva Santé Animale, Duesseldorf, Germany; c Clinic for Swine at the Centre for Clinical Veterinary Medicine, Ludwig-Maximilians-University Munich, Oberschleissheim, Germany; d IVD Society for Innovative Veterinary Diagnostics mbH, Seelze-Letter, Germany; e Vaxxinova diagnostics GmbH, Leipzig, Germany

**Keywords:** Swine influenza A virus, zoonosis, porcine respirovirus 1, swine orthopneumovirus, emerging viruses, surveillance, multiplex RT-qPCR

## Abstract

Respiratory disease is a significant economic issue in pig farming, with a complex aetiology that includes swine influenza A viruses (swIAV), which are common in European domestic pig populations. The most recent human influenza pandemic in 2009 showed swIAV’s zoonotic potential. Monitoring pathogens and disease control are critical from a preventive standpoint, and are based on quick, sensitive, and specific diagnostic assays capable of detecting and distinguishing currently circulating swIAV in clinical samples. For passive surveillance, a set of multiplex quantitative reverse transcription real-time PCRs (mRT-qPCR) and MinION-directed sequencing was updated and deployed. Several lineages and genotypes of swIAV were shown to be dynamically developing, including novel reassortants between human pandemic H1N1 and the avian-derived H1 lineage of swIAV. Despite this, nearly 70% (842/1216) of individual samples from pigs with respiratory symptoms were swIAV-negative, hinting to different aetiologies. The complex and synergistic interactions of swIAV infections with other viral and bacterial infectious agents contribute to the aggravation of pig respiratory diseases. Using a newly developed mRT-qPCR for the combined detection of swIAV and the recently described porcine respirovirus 1 (PRV1) and swine orthopneumovirus (SOV) widespread co-circulation of PRV1 (19.6%, 238/1216 samples) and SOV (14.2%, 173/1216 samples) was evident. Because of the high incidence of PRV1 and SOV infections in pigs with respiratory disease, these viruses may emerge as new allies in the porcine respiratory disease syndrome.

## Introduction

Respiratory disease is one of the most common challenges in pig production. The complex aetiology involves physico-chemical stressors and both viral and bacterial agents. The associated clinical signs, characterized by coughing with or without fever especially in nurseries, do not allow an aetioligical diagnosis. One of the most common problems in pig farming is respiratory disease. The multifaceted aetiology includes physicochemical stresses as well as viral and bacterial pathogens. The related clinical indicators, which include coughing with or without fever, are insufficient to make an aetioligical diagnosis, particularly in nurseries. Apart from swine influenza A virus (swIAV), several other negative-stranded RNA viruses have recently been added to the list of potential porcine respiratory pathogens. These comprise the recently discovered porcine respirovirus 1 (PRV1, formerly known as porcine parainfluenza virus) and swine orthopneumovirus (SOV) [1,2]. Their role, if any, in the porcine respiratory disease complex (PRDC) remains to be defined.

SwIAVs, in contrast, are well known to play an integral part in PRDC, pathing ways for further opportunistic agents and aggravating clinical signs of co-infections [3]. It is described that in sows, swIAV infections can lead to reproductive disorders like return to estrus, abortions and feeble piglets most likely as a result of short-lived bouts of high fever [4–6]. Three major influenza A virus (IAV) subtypes (H1N1, H1N2, and H3N2) with numerous genotypes and variants have been identified in European pig populations so far [7–13]. Intensive pig production in Europe becomes dominated by large compounds that continuously produce high numbers of piglets in weekly cycles with up to several thousands of sows per farm. Along with that transition in swine population structure, away from small, clustered, family-owned swine farms the dynamics of swIAV infections in European pig herds started shifting. Instead of short, epizootic, acute, and self-limiting outbreaks of respiratory disease, a self-perpetuating state of infection (enzootic infection) of the affected farms becomes increasingly widespread. The latter is characterized by smoldering respiratory symptomatology in piglets and fattening pigs and persistent fertility problems in sows lasting for months and even years [14–16]. As a result, in contrast to purely seasonal influenza in humans, swIAV can be present in European pig farms all year [9]. This consistently compromises animal wellbeing, causes economic losses, and increases zoonotic risk. Several genetic building blocks linked with higher zoonotic potential have been discovered in European swIAV, which could result in the establishment of a highly zoonotic strain in the event of forced reassortment [9,17–20]. In today’s herds, the genetic and antigenic features of circulating swIAV are gradually diversifying, and a multitude of novel reassortant viruses have developed from the co-circulation of distinct lineages of the main swIAV subtypes. Sustained swIAV replication in closed, large farms is associated with accelerated antigenic drift [16,21]. These developments challenge routine diagnosis by real time RT–PCR (RT-qPCR) as well as prevention and control strategies based on licensed but also autologous vaccines.

Respiroviruses of the Paramyxoviridae family have historically been linked to respiratory diseases in humans and other animal species [18,22,23]. They were recently described as a new virus in pigs that was first detected in 2013 in swab samples of pigs that died spontaneously at a slaughterhouse in Hong Kong, China [24]. In the follow-up, PRV1 was detected in the United States in 2016, Chile (2015-2019), Poland (2019-2020), and, as of 2020, Hungary, Germany, and the Netherlands [24–29]. Based on limited sequencing data, two separate clades were discovered, with one European and one Hong Kong sequence (clade 1) and one American and three Asian sequences (clade 2) [30]. Little is known about the epidemiology and clinical impact of PRV1 in the frame of PRDC. Despite the fact that PRV1 is genetically related to human respirovirus, its zoonotic potential is unknown [31,32].

In parallel, an orthopneumovirus (tentatively referred to as SOV) was discovered in feral pigs in the United States in 2016 using metagenomic sequencing of nasal swabs [1]. Decades ago, in 1998, antibodies that cross-reacted with the bovine respiratory syncytial pneumovirus (BRSV) were found in serological surveys of pigs in Ireland, despite the fact that no corresponding virus was found [33]. In response to the recent discoveries in the United States, a seroprevalence research in France revealed the presence of this virus in pigs, possibly in conjunction with respiratory disorders [34]. Further research found SOV in Spain in 2022 [35]. SOV has not yet been isolated, and its prevalence and pathogenicity are unclear. However, several ortho- and metapneumoviruses have been discovered as significant respiratory infections of farm animals and humans [36–38]. Monitoring of swIAV is pivotal from an OneHealth preventive perspective. This is based on rapid, sensitive and specific multiplexed real-time RT–PCR (mRT-qPCR) diagnostic assays fitted here to detect and discriminate currently circulating swIAV in clinical samples. PRV1 and SOV have not yet been included in routine diagnostic algorithms of PRDC. Along with a continued update of swIAV surveillance in pigs in Germany, we therefore developed, conducted performance studies and used m RT-qPCRs for the detection of PRV1 and SOV.

## Material and methods

### Reference viruses and field samples

Viral RNA of reference swIAV strains from the inventory of the National Reference Laboratory for Avian Influenza Virus at the Friedrich-Loeffler-Institute (FLI) were used to characterize test performance of the modified multiplex swIAV-subtyping RT-qPCRs (triplicate analyzes). In addition to submissions from former studies [9], nasal swab samples derived from pigs with respiratory disease were obtained from German pig holdings and from external diagnostic laboratories since 2020. Samples were submitted cooled in viral transport medium (SIGMA VIROCULT®, MWE) to FLI.

Samples received (n = 1,216 from 123 swine holdings; supplemental Table 1) were analyzed with the use of established and newly developed/adapted RT-qPCRs assays [39]. For positive samples with cycle of quantification (cq) values <30, virus isolation in Madin-Darby-Canine kidney cells (MDCK-II), MDCK sialytransferase-supplemented cells (SIAT1) or swine testicle (ST) cell cultures was attempted. Depending on the cq value, original samples or isolates thereof were subsequently examined by full-length nucleotide sequence analyzes of the HA genome segments or the full genome.

Other porcine respiratory pathogens of viral and bacterial nature were used for assessing analytical specificity of newly developed assays (supplemental Table 2).

### Viral RNA extraction

Viral RNA was either extracted by using the QIAamp Viral RNA Mini Kit (Qiagen, Hilden, Germany) from 140 µl volume of each field sample (nasal swab, oral fluid or lung homogenate supernatant) or by using 100 µl volume within the NucleoMag®VET Kit (Macherey-Nagel GmbH & Co. KG, Dueren, Germany) according to the manufacturer’s instructions and stored at −20°C until use.

### Design of primers and probes

Primers and probes for modifying subtype and/or lineage-specific detection of fragments of the swIAV HA (hemagglutinin) and NA (neuraminidase) genome segments by use of mRT-qPCRs were based on previous assays [39] or manually selected from HA and NA alignments comprising a selection of recently circulating swIAV (this study) as well as current sequences of Eurasian origin (GenBank at NCBI, EpiFlu, Global Initiative on Sharing All Influenza Data (GISAID), Influenza Research database (IRD)). Assays were designed to detect and discern the main porcine HA subtypes H1av (clade 1C), H1pdm (clade 1A), H1hu (clade 1B) and H3. The occurrence of a recent spill-over of a human seasonal H3-subtype (H3hu 2004/2005-derived) into the swine population gave need for the selection of further HA-differentiating primers and probes [40–42].

Primers and probes for detection of PRV1 were selected from each the fusion (F), the nucleoprotein (NP) and the phosphoprotein (P) gene by aligning available sequences from all databases.

For the characterization of SOV, sets of primers and probes were designed based on alignments of NP, G and M gene sequences found in databases.

Using the online tool “Oligocalc,” melting temperatures and basic properties of all oligonucleotides were approximated [43]. Final sets of primers and probes for RT-qPCR are listed in supplemental Tables 3–5.

### Multiplex RT-qPCRs

Twenty-five µL per reaction (including 5 µL of template RNA) were prepared using the AgPath-ID^TM^ One-Step RT-qPCR kit (Thermo Fisher Scientific, United States) with AmpliTaq Gold DNA Polymerase and the following temperature profiles on a Biorad CFX96 Real-Time cycler (Biorad, Germany) and corresponding collection of fluorescent signals FAM, HEX, ROX and/or CY5, respectively, during the annealing phase:

- *Multiplex-swIAV-subtyping assays:* Reverse transcription at 45°C for 10 min, initial denaturation at 95°C for 10 min, 42 cycles of PCR amplification at 95°C for 15 s, 58°C for 30 s, respectively, and 72°C for 30 s in a 20 µl reaction mixture using optimized concentrations of forward and reverse primers and probes.
- *Triple-pathogen (swIAV-PRV1-SOV) assay:* Reverse transcription at 45°C for 10 min, initial denaturation at 95°C for 10 min, 42 cycles of PCR amplification at 95°C for 15 s, 56°C for 20 s, and 72°C for 30 s in a 20 µl reaction mixture using optimized concentrations of forward and reverse primers and probes.

Amplification data were analyzed with the Bio-Rad CFX Manager software.

The specificity of the assays was evaluated with viral RNA from representative swIAV reference viruses that had been subtyped based on full-length sequence analysis (Table 1) or, for the triple-pathogen assay, from positive and sequenced controls generated from field samples within this study. In addition, different IAV subtypes and IAV of other host species (avian and human influenza viruses) and other porcine viral and bacterial respiratory pathogens were used. By testing 10-fold serial dilutions of viral RNA extracted from representative viruses, detection limits (LOD) were determined based on triplicate analyzes and, for the swIAV-subtyping assays, compared to the modified IAV generic M gene-specific RT-qPCR [39]. The threshold distinguishing positive from negative reactions was set at cq 40, values ≤ 39.9 were considered as positive.

**Table 1.** Analytical performance of primers and probes for detection and differentiation of HA and NA subtypes/lineages of swine influenza A viruses from European pig holdings.

Isolate identification	Lineage		RTqPCRs (Cq-values)								
	HA clade	NA	*M*	Hemagglutinin (HA)					Neuraminidase (NA)		
				H1pdm^a^	H1hu^a^	H3hu^a^	H1av^b^	H3sw^b^	N1all^c^	N1pdm^c^	N2^c^
A/swine/Germany/AR2013/2015	1C	N1av	15.47	Neg	neg	neg	16.40	neg	16.05	neg	neg
A/swine/France/AR1123/2015	1C	N1av	14.57	Neg	neg	neg	15.75	neg	16.28	neg	neg
A/swine/Denmark/AR570/2016	1C	N2	15.03	neg	neg	neg	16.79	neg	neg	neg	15.98
A/swine/France/SIR3052/2017	1B	N2	15.30	neg	15.49	neg	neg	neg	neg	neg	16.88
A/swine/Spain/AR1297/2016	1B	N2	20.14	neg	20.63	neg	neg	neg	neg	neg	21.31
A/swine/Netherlands/AR647/2015	1B	N1av	19.83	neg	19.75	neg	neg	neg	20.13	neg	neg
A/Germany-NDS/14/2007	H1	N1	30.12	neg	33.41	neg	neg	neg	30.58	neg	neg
A/Wild duck/Germany/R30/2006	H1	N1	25.81	neg	neg	neg	neg	neg	24.53	23.91	neg
A/swine/England/SIR2972/2017	1A	N1pdm	17.20	16.36	neg	neg	neg	neg	neg	16.38	neg
A/swine/Republic of Ireland/SIR2389/2017	1A	N1pdm	14.57	15.41	neg	neg	neg	neg	neg	14.31	neg
A/swine/Germany/AR8097/2016	1A	N2	18.41	18.60	neg	neg	neg	neg	neg	neg	18.51
A/swine/Denmark/SIR1570/2017	1A	N2	14.91	16.07	neg	neg	neg	neg	neg	neg	15.31
A/swine/Serbia/SIR4880/2017	1A	N1av	16.78	16.56	neg	neg	neg	neg	15.87	neg	neg
A/swine/Germany/SIR5321/2017	H3	N2	15.38	neg	neg	neg	neg	16.11	neg	neg	16.01
A/swine/Netherlands/AR531/2015	H3	N2	15.28	neg	neg	neg	neg	16.76	neg	neg	15.76
A/swine/Denmark/SIR1299/2017	H3hu	N2	15.28	neg	neg	16.76	neg	neg	neg	neg	15.54
A/swine/Germany/Bak20/2016	H3hu	N2	13.15	neg	neg	13.38	neg	neg	neg	neg	14.39
A/Waterfowl/Germany/2311/2009	H3	N8	24.7	neg	neg	neg	neg	neg	neg	neg	neg

No other viral (list) or bacterial agents (list) associated with PRDC gave positive signals in any of these PCRs (supplemental Table 3).

^a^ RT-qPCR is compound of triplex HA mRT-qPCR.

^b^ RT-qPCR is compound of duplex HA mRT-qPCR.

^c^ RT-qPCR is compound of NA triplex mRT-qPCR.

### Conventional one step RT-PCR and sequencing of swIAV

Sequences of the HA IAV-gene from samples with cq values ranging from 30 to 34 were generated by Sanger sequencing after conventional RT–PCR amplification with Superscript III Reverse Transcriptase One-Step RT–PCR kit with Platinum Taq polymerase (Invitrogen^TM^ GmbH, Karlsruhe, Germany) in a volume reaction of 25 µL, including 5 µl of template RNA. Primers for amplification of the full length HA gene or overlapping fragments thereof have been described recently [44–46]. Thermocycling conditions on an Analytik Jena Flex Cycler were optimized by adapting annealing time and temperature: 50°C 30 min, 94°C 2 min, 10 cycles each of 94°C 30 s, 50°C 10 s –72°C 20 s, 30 cycles 94°C 30 s, 56°C 1 min 72°C – 5 min, final elongation 72°C 5 min. Specific amplicons were purified from 1.5% agarose gels using a QIAquick gel extraction kit (Qiagen, Hilden, Germany). The sequences were analyzed on an ABI310 sequencer, and assembled using the Geneious software version 2021.0.1. Generated sequences were screened on IRD or GISAID databases with BLASTN2 to identify closely related sequences.

Selected field samples with cq values < 30 were subjected with prior amplification to full genome sequencing by nanopore technology as previously described [47]. Sequences were deposited in the EpiFlu database (www.gisaid.org).

### Genotyping and phylogenetic analyzes

Genotyping of the internal genome segments (IGS) PB2, PB1, PA, NP, M and NS, was achieved by aligning full length segmental swIAV sequences obtained by nanopore-directed sequencing of clinical samples and/or virus isolates with reference sequences of avian-derived (av, clade 1C) and pandemic (pdm, clade 1A) H1N1 subtype sequences. Neighbor-joining distance driven analyzes allowed a dichotomus designation to either of the lineages. In a similar approach, the neuraminidase sequences were assigned to subtypes N1 and N2, and within the subtypes to lineages N1av, N1pdm, N2s and N2g.

Deduced amino acid HA sequences were subjected to phylogenetic analyzes. A maximum likelihood approach (IQ-Tree [48]) was employed utilizing ModelFinder [49] and an ultrafast bootstrap approximation method [50].

## Results

### Genetic drift in swIAV sequences required adaptation of primer and probe sequences of mRT-qPCRs for swIAV subtype characterization

**Figure 1.**
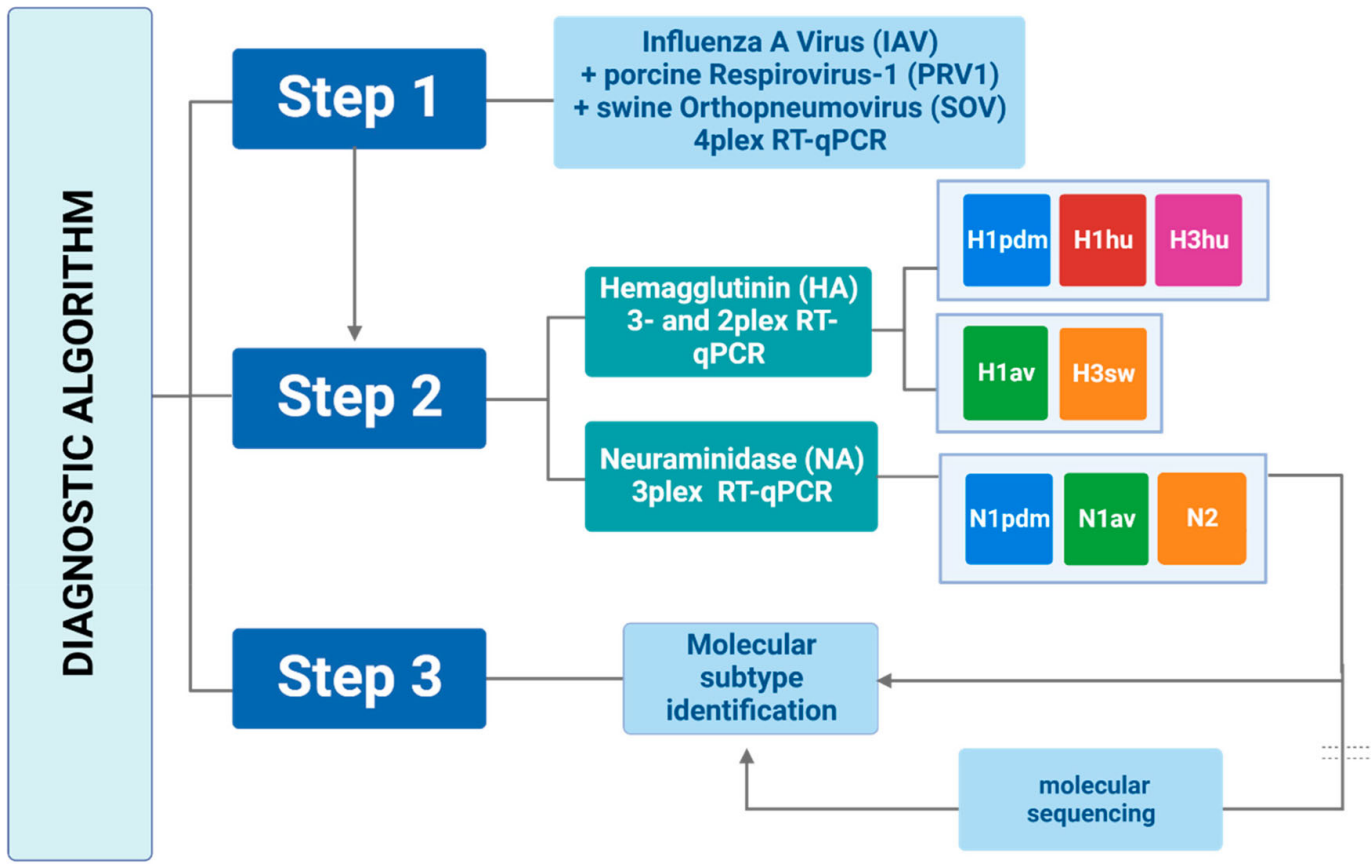
Diagnostic algorithm based on one-step multiplex RT-qPCRs for detection and subtyping of swine influenza A viruses (swIAV) as well as the detection of porcine respirovirus 1 and swine orthopneumovirus circulating in European pig populations. Step 1 depicts a tetraplex RT-qPCR, targeting the M-gene segment of (sw)IAV, the F-gene of PRV1 and the NP-gene of SOV; an internal control (IC2) is essentially included in this tetraplex RT-qPCR (not shown). In step 2, subtyping for IAV RNA-positive samples is attempted employing the one-step duplex- and triplex HA- and the triplex NA-specific RT-qPCRs developed in this study. Step 3 is only required in case if HA or NA subtype/lineage cannot be assigned by the shown RT-qPCRs: HA and/or NA amplicons need to be generated by conventional one-step RT-PCR for Sanger amplicon or minION sequencing and BLAST searches or swine H1 clade classification by Anderson, Macken [57] on the Influenza Research Database (IRD) to finalize subtyping of swIAV.

Extensive *in silico* analysis showed that significant sequence variation within the HA and NA of European swIAV subtypes and lineages has accumulated over the last years (data not shown). This has caused mismatches in primers and probes in several positions of five HA and three NA targets that were composed into two triplex- and one duplex amplification assays (supplemental Table 2, red coloured nucleotides). The triplex HA RT-qPCR differentiated two H1 lineages human pandemic H1 [H1pdm, clade 1A, FAM] and human seasonal H1 [H1hu, clade 1B, ROX] as well as the human-derived H3 subtype [H3hu, Cy5] (Figure 1). The duplex HA RT-qPCR detected avian-origin porcine H1 [H1av, clade 1C, HEX] as well as the porcine H3 subtype [H3, Cy5]. Differentiation of N1 and N2 subtypes was attempted by generating broadly reacting RT-qPCRs for subtypes N1 [N1all, FAM], human pandemic/2009 N1 [N1pdm, ROX] and N2 [HEX] in a triplex RT-qPCR. Thus, N1pdm positive samples give positive results for both N1 RT-qPCRs (supplemental Table 1). RNA obtained from reference swIAV was used to evaluate the sets of adapted primers and probes for their analytical specificity for the different lineages of European swIAV (Table 1). In addition, pre-selected M-RT-qPCR-positive samples (clinical samples, field specimens and isolates), derived from German pig populations with overt respiratory symptoms, were tested in order to evaluate the diagnostic performance capacity of the modified mRT-qPCR assays (supplemental Table 2). IAV of other host species and subtypes and other porcine viral and bacterial respiratory pathogens tested were not detected by any of the specific RT-qPCRs, thus showing 100% analytical specificity (supplemental Table 3).

As shown in Table 1, the newly designed and modified oligonucleotide sets sharply discriminate between the different subtypes and lineages. Highly lineage- and subtype-specific detection with no cross-reaction was evident even in samples with very high virus loads. Except for samples with low viral loads of cq > 34, HA and NA subtypes could be assigned by subtype-specific mRT-qPCRs to nearly all IAV-positive samples tested. These mRT-qPCRs did not yield positive signals for any of the analyzed IAV-negative samples (data not shown). Comparison of cq values of serial 10-fold RNA dilutions with results of the generic M-specific RT-qPCR was used to assess the relative analytical sensitivity of the mRT-qPCRs. In general, detection limits of the swIAV mRT-qPCRs were very similar to those of the corresponding M-specific RT-qPCR (Table 2).

**Table 2.** A–C. Relative sensitivity of (A) triplex hemagglutinin, (B) duplex hemagglutinin and (C) triplex neuraminidase-specific RT-qPCRs compared with IAV-generic matrix-specific amplification.

(A)						
RNA dilution	3plex HA RT-qPCR (Cq-values)					
	*M*	H1pdm	*M*	H1hu	*M*	H3hu
0	23.29*	22.75	24.47	23.77	24.37	24.35
−1	26.36	25.91	27.74	27.13	27.86	27.52
−2	29.63	29.74	31.06	31.23	31.15	30.95
−3	32.61	33.16	34.47	33.19	34.36	33.04
−4	36.25	37.42	37.84	37.45	37.69	36.67
−5	Neg	neg	neg	neg	neg	neg
−6	Neg	neg	neg	neg	neg	neg

(B)				
RNA dilution	2plex HA RT-qPCR (Cq-values)			
	*M*	H1av	*M*	H3(sw)
0	25.04	25.36	25.03	24.02
−1	28.32	28.11	28.40	28.42
−2	31.60	31.01	31.68	30.60
−3	35.02	34.36	35.24	33.65
−4	39.91	37.02	38.48	37.11
−5	Neg	neg	neg	neg
−6	Neg	neg	neg	neg

(C)						
RNA dilution	3plex NA RT-qPCR (Cq-values)					
	*M*	N1all	*M*	N1pdm	*M*	N2
0	25.04	24.85	23.29	21.15	24.02	24.90
−1	28.32	28.12	26.36	24.18	28.42	28.14
−2	31.60	31.19	29.63	27.77	30.60	31.68
−3	35.02	34.42	32.61	30.35	33.65	35.31
−4	39.91	37.51	36.25	34.32	37.11	38.71
−5	Neg	N/A	neg	neg	neg	neg
−6	Neg	neg	neg	neg	neg	neg

*All values represent means of triplicate runs.

In order to mimic co-infections with different swIAV lineages/subtypes, the RNA of each two viruses was mixed in approximately equal amounts (in 95:5, 50:50 and in 5:95) to mimic an up to 20-fold difference in RNA content. Cq values of the M-specific RT-qPCR were used to normalize the concentration of viral RNA in advance, assuming that this PCR-amplified viral RNA of the different subtypes/lineages with similar efficacy. All assays were able to detect and differentiate both HA and NA targets in the sample in all mixtures, and no cross-reactivity to lineages not represented in the sample mix was evident (supplemental Table 5).

mRT-qPCRs for subtyping of swIAV have been updated and adapted to guarantee that for each sample, IAV positive with cq values <34 by generic M gene-specific RT-qPCR, both HA and NA subtypes could be determined.

### Continuing diversifying evolution and new reverse zoonotic introductions of human pandemic H1N1 shaped swIAV populations in Germany since 2019

**Figure 2.**
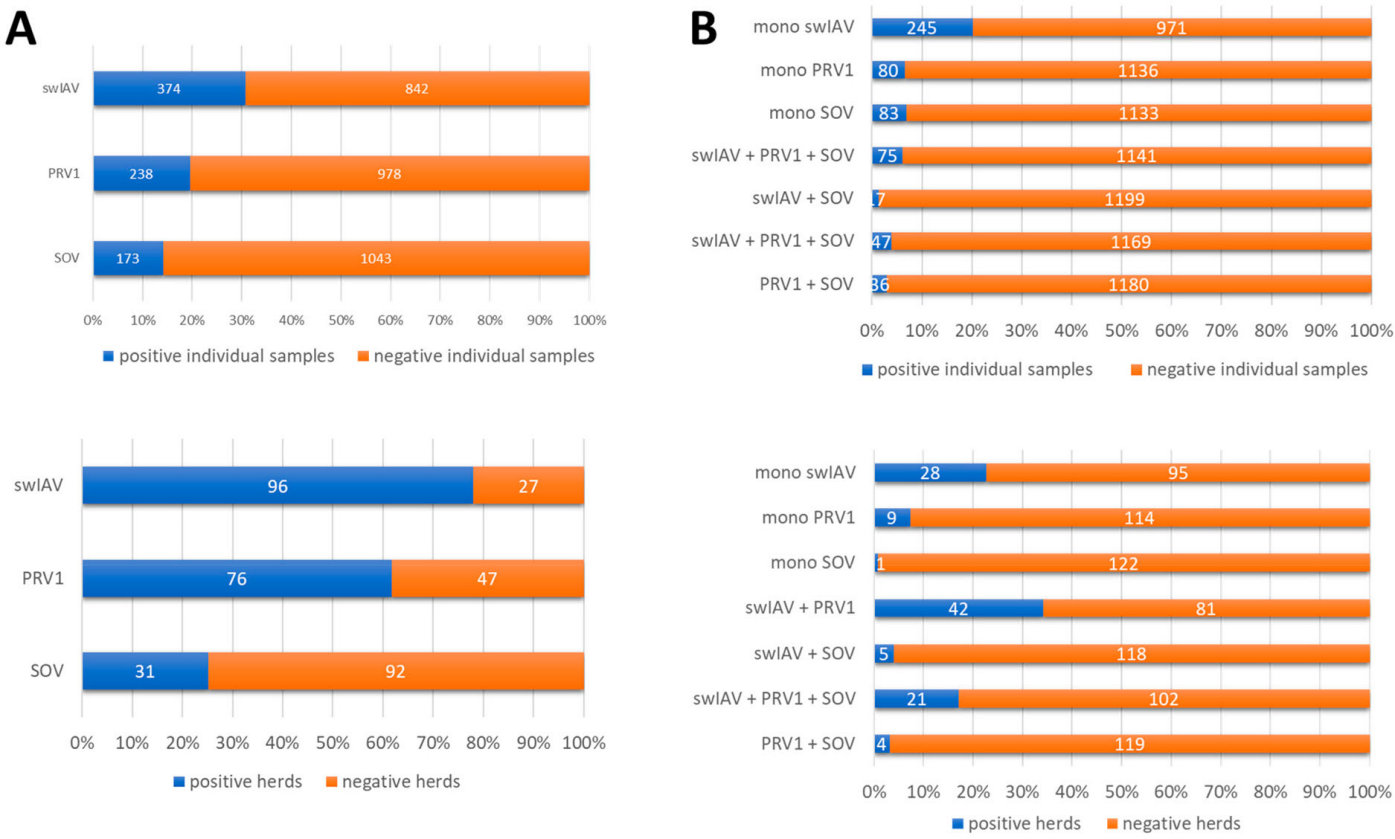
(A) Total detection of swIAV, PRV1 and/or SOV infection and (B) stratification of mono-, di- and triple-infections in individual clinical samples of domestic pigs with respiratory signs of disease and pig herds in Germany from April 2021 to August 2022.

**Figure 3.**
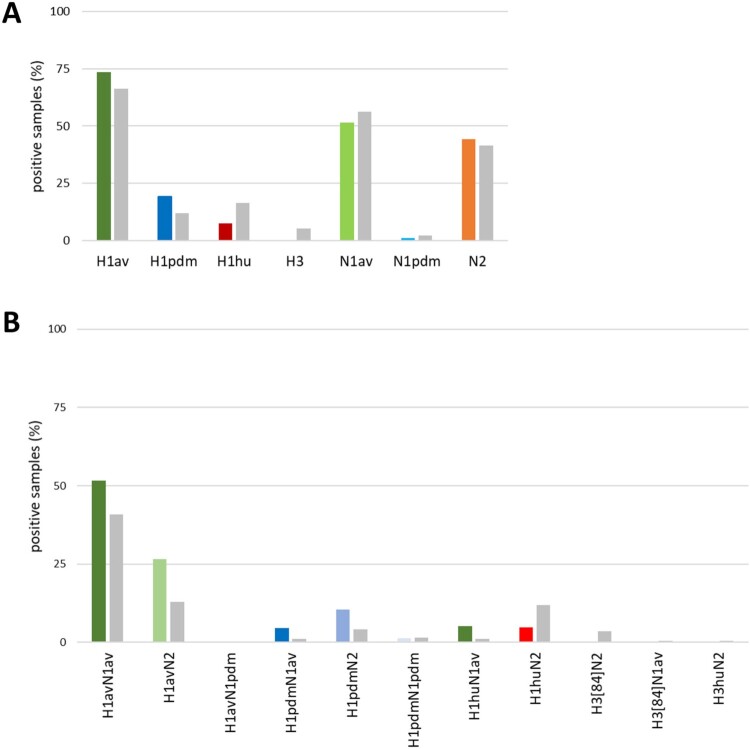
Frequency of detection of swIAV infection in European domestic swine with respiratory symptoms from April 2021 to August 2022 and stratified by (A) HA and NA subtypes separately, and (B) combined HA and NA subtyping for samples (coloured bars) in comparison to the results from the study of Henritzi et al., 2020 (grey bars) [9]. Viruses of the H3 subtype were not detected in the period of investigation (2021–2022).

Screening for IAV revealed 30.8% of the individual samples (374/1,216) to be positive and 78.1% of the farms (96/123) to be infected (Figure 2(A)). Findings included all three main H1-clades (1A-1C) of swIAV as well as several reassortants among them (Figure 43). In line with the study of Henritzi et al. [9], increased detection of clade 1C (73.4%) and its reassortants continued, followed by clade 1A (19.1%). Clade 1B (7.6%) and HA subtypes H3 and H3hu were further declining in frequencies or (H3) not detected at all (Figure 3(A)). Concerning the NA segment, the dominating subtype was N1av (51.6%), followed by N2 (44.4%) and N1pdm (1.0%) (Figure 3(A)). In summary, subtype H1avN1av was detected most frequently, followed by subtype H1avN2 and then H1pdmN2 and H1pdmN1av, respectively (Figure 3(B)).

Amplicon sequencing based on either the pan-HA RT-PCRs or Nanopore sequencing technology described by King et al. (2020) was used to verify subtyping results generated by mRT-qPCRs and to provide sequence data for phylogenetic analyzes. However, some isolates and clinical samples failed to yield reliable HA sequences (supplemental Table 5, “questionable”). Finally, a harmonized diagnosis could be made by combining the results of mRT-qPCR and sequencing: In all cases for which results were available for both methods, fully concordant subtyping results were obtained for both HA and NA (supplemental Table 7). However, in a few HA samples, where the updated pan-HA primer set and Nanopore technology failed to generate an amplicon, mRT-qPCRs could assign the subtypes.

Concerning tested field samples, only in few cases mRT-qPCRs detected the presence of swIAV-mixtures/co-infections with subtypes H1 clades 1A-C as well as N1 and N2 subtypes in the same sample (supplemental Table 2).

### Phylogenetic analyzes of currently circulating German swIAVs reveal novel genotypes

**Figure 4.**
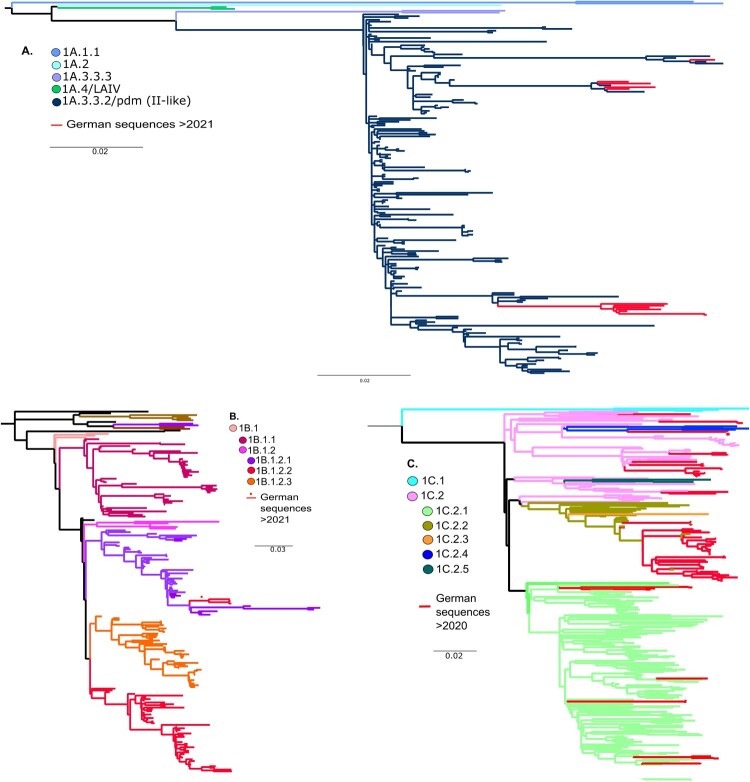
**(A)** Phylogenetic tree of swine H1 HA genes of the 1A-lineage (H1pdm) coloured by clade and annotated by global H1-lineage nomenclature by Anderson et al. (2016). Analyzes were conducted with established sequences of collected samples within this study (2021–2022) and reference sequences and data accessible on GISAID or shared via the OFFLU swine IAV working group. **(B)** Phylogenetic tree of swine H1 HA genes of the 1B-lineage (H1hu) coloured by clade and annotated by global H1-lineage nomenclature by Anderson et al. (2016). Analyzes were conducted with established sequences of collected samples within this study (2021–2022) and reference sequences and data accessible on GISAID or shared via the OFFLU swine IAV working group. **(C)** Phylogenetic tree of swine H1 HA genes of the 1C-lineage (H1av) coloured by clade and annotated by global H1-lineage nomenclature by Anderson et al. (2016). Analyzes were conducted with established sequences of collected samples within this study (2021–2022) and reference sequences and data accessible on GISAID or shared via the OFFLU swine IAV working group.

A total of 105 samples (either nasal swabs or cell-culture-derived virus isolates) were selected for whole genome sequencing. Separate phylogenetic trees for the HA segment were built by maximum likelihood analyzes [48]. All recent German clade 1A sequences (*n* = 12) generated within our study since 2021 clustered in clade 1A.3.3.2/pdm (II-like) (Figure 4(A)). A total of three clade 1B (H1hu) sequences from 2021 to 2022 lined up in clade 1B1.2.1 of the human seasonal lineage (Figure 4(B)). This lineage holds all H1hu sequences from Germany. Phylogenetic analyzes performed for clade 1C viruses (n = 90) identified four genetic subclades. Most of the German samples clustered into clade 1C.2.2 (*n* = 50), 1C.2 (*n* = 22), 1C.2.1 (*n* = 13), and clade 1C.2.4 (*n* = 5) (Figure 4(C)). Among the 1C viruses an increase in frequency of the Danish origin subtype H1avN2, here clustering with clade 1C.2, was evident since 2020 (Figure 3(B)).

For a total of 64 swIAVs-positive samples full length genome sequences were obtained and used for genotype analyzes (Table 3). In combination with the HA and NA subtype, 62 isolates could conclusively be assigned to a total of 15 genotypes. 10 genotypes among these had been already described [9], whereas five genotypes are novel (AQ, AR, AS, AT and AU in Table 3). In 46.9% of the genotyped swIAV, exclusively avian-derived internal genome segments (IGSs) were present, whereas 12.5% revealed IGSs of H1pdm origin. All others (40.6%) were constituted reassorants of various avian and pandemic IGSs.

**Table 3.** Genotyping of full length genome segments of 64 swIAV isolates employing the nomenclature of Henritzi et al. [9].

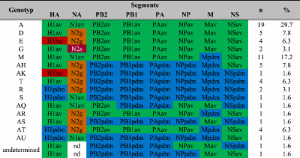

The lettering indicates phylogenetically different lineages for each segment. Internal gene segments PB2 to NS are distinguished by avian (av; green colour) or human pandemic 2009 (pdm, blue) origin. N2g and N2s indicate relationship with A/sw/Gent/1/1984-like or N2s – A/sw/Scotland/410440/1994-like viruses.

### High incidences of PRV1 and SOV and co-infections with swIAV infections in pig herds with acute respiratory disease in Germany

Due to the surprisingly high number (69.2%, 842/1216) of swIAV-negative individual samples of pigs despite showing acute respiratory signs, the material was examined for the putative viral pathogens PRV1 and SOV, that were recently identified in the frame of respiratory disease in pigs.

On basis of limited sequencing data available in public databases, we established RT-qPCRs for PRV1 and SOV (Figure 1, supplemental Tables 6 and 7, supplemental Figure 1). In the absence of reference sequences based on virus isolates, relative sensitivity of different primer/probe sets covering different viral genes was tested by using 10-fold serial dilutions of RNA positive field samples for the corresponding viruses. Amplificates obtained with the positive field samples have been Sanger sequenced to confirm the specificity; due to the very short resulting sequences these were not deposited in a public database. In comparison to oligonucleotides covering the NP and P gene, the RT-qPCR targeting the F-gene of PRV1 revealed to be most sensitive and were used in the following (data not shown). For SOV, a combination of primers and probes covering two non-overlapping regions of the NP gene as well as targets on the M and G gene were screened. The NP-specific RT-qPCR revealed greater sensitivity (data not shown).

For simultaneous detection of three pathogens in the same tube, the IAV generic M-specific RT-qPCR [39] (FAM) was combined with the PRV1 F- (ROX) and SOV NP-specific assays (Cy5), together with a heterologous internal control system IC2, based on a fragment of a gene encoding EGFP (HEX, see Figure 1 [51]). Results shown in Figure 2 (and supplemental Tables 2 and 3) prove full analytical specificity excluding several porcine viral and bacterial respiratory pathogens. Analytical performance of the individual PCRs was not affected by combining all three mixes (data not shown). Relative sensitivity of this mRT-qPCR was tested by using 10-fold serial dilutions of RNA extracted from field samples positive for the corresponding virus (Table 4), proving very similar analytical sensitivity to the validated M-specific RT-qPCR.

**Table 4.** Relative sensitivity of the established triple-pathogen RT-qPCR specific for swine influenza A virus, porcine respirovirus 1 and swine orthopneumovirus, respectively.

RNA dilution	3plex RT-qPCR (Cq-values)		
	PRV1	SOV	M
0	23.56	22.86	23.29
−1	27.62	26.10	26.36
−2	31.00	29.31	29.63
−3	34.16	32.68	32.61
−4	37.58	35.27	36.25
−5	neg	neg	Neg
−6	neg	neg	Neg

The PRV1 screening revealed the presence of 19.6% positive samples (238/1216) with varying viral loads (ranging from cq 20-38; supplemental Table 2) and 61.8% of the farms (76/123) to be PRV1 infected (Figure 2(A)). For SOV, 14.2% (173/1216) of the samples tested gave positive results (Figure 2) and 25.2% (31/123) of the farms were infected.

A total of 6.6% (80/1216) of PRV1 and 6.8% (83/1216) of SOV positive samples tested positive only for PRV1 and SOV, respectively. Looking on co-infections, 7.2% (75/1216) of the PRV1 positive samples were associated with swIAV positive results, and 3% (36/1216) with SOV co-infection (Figure 2(B)). Co-infections of SOV with swIAV were detected in 1.4% (17/1216) of the nasal swabs from diseased pens. Except two samples with high viral loads (cq values: 19-20), all other SOV samples revealed low viral loads (cq >30; supplemental Table 2). In contrast to swIAV and PRV1, few SOV positive herds were found, but within these herds, a larger number of animals appeared to be SOV positive.

In an additional 3.9% of the samples (47/1216), we were able to detect triple infections with swIAV, PRV1 and SOV (Figure 2(B), supplemental Table 2).

Altogether, our results show that besides swIAV PRV1 is widely spread in Germany. PRV1 positive holdings in contrast to SOV-positive ones were more frequently associated with swIAV.

## Discussion

Despite the fact that the polymicrobial nature of the porcine respiratory disease complex (PRDC) is a well-established and widely acknowledged concept, the participation, contribution, and interaction of diverse pathogens in that complex is still unknown. The accentuated role of swIAV has long been established, however, these viruses remain highly mobile targets that are notoriously difficult to diagnose due to their remarkable genetic flexibility. Recently, two new putative viral players have been detected in this field: porcine respiro- (PRV1) and swine orthopneumoviruses (SOV) [1,2,23,24,26–28,33–35,52].

Increasing pig herd size and changing infrastructures were predicted to create new niches fostering enzootic virus circulation and enforced emergence at least of swIAV [13]. The most recent human influenza pandemic in 2009 revealed the potential impact of swIAV in terms of causing pandemics, emphasizing the importance of ongoing swIAV surveillance. Despite the fact that swIAV has zoonotic and even (pre)pandemic potential, there is no ongoing government-managed surveillance programme in place to monitor swIAV in European pig populations. SwIAV infection can be controlled by biosecurity, herd management and vaccination. Increased understanding of within-herd viral dynamics and evolution is required to optimize intervention and prevention approaches that address compromised animal welfare, ongoing productivity losses, and public health threats. Within this framework, mRT-qPCRs were developed, enabling for a time-efficient and cost-effective assessment of three viral porcine respiratory pathogens in a single, updated approach with the goal of maximizing inclusiveness and specificity. Analytical specificity testing of the primers and probes employed in these mRT-qPCRs validated their swIAV-lineage- and pathogen-specific reactivity. Thus, co-infections with various swIAV-lineages, as well as up to triple infections with swIAV, PRV1, and SOV in the same field sample, were detected with high reliability. Even samples with low swIAV-specific RNA content (cq values >33) could be subtyped, demonstrating the mRT-qPCRs’ significant benefits over previously employed amplicon sequencing technologies. In line with former studies, increasing diversity of HA/NA reassortant patterns, especially within the H1 subtype were found, while no longer representatives of the H3 subtype were detected [11,53–55]. While H1 clade 1C viruses continue to predominate, our analysis found an increase in clade 1A viruses as well as a minor rise in subtype N2 in contrast to Henritzi et al. [9]. Subtypes H1hu (1B) and H3 are becoming less common. It’s worth noting that the original 1C NA segment of human pandemic H1N1 viruses in pigs has been nearly entirely replaced by 1C N1 or N2. The previously observed strong reassortment activity between the 1A and 1C swine lineages has resulted in the creation of new genotypes. This produced further swIAV strains harbouring IGSs of the pandemic 2009 virus but expressing HA and NA proteins distinct from this human virus. It remains to be determined whether and how this affects zoonotic propensity of these viruses. There is an intimate interface between pigs and men, and swine were associated with the root of the last human influenza pandemic [56]. An important future objective of swIAV investigations therefore should also focus more intensively on the characterization of the zoonotic propensity of these viruses.

Despite considering that just a few PRV1 and SOV whole genome sequences from Europe are available, we developed laboratory protocols that are shown here to detect and identify swIAV, PRV1, and SOV simultaneously. The existence of PRV1 and SOV in German pig populations has recently been demonstrated, and this evidence is expanded here [27]. Previous research found swIAV together with PRV1 and SOV in Spanish pig nurseries [52], and the discovery of PRV1 in Hungary, Poland, and the Netherlands suggests that the virus is widespread in Europe. However, no data from other major pig-producing countries are currently available. Interestingly, swIAV and PRV1 co-infections were more frequently detected than swIAV and SOV co-infected samples and fewer SOV infected premises were identified compared to swIAv and PRV1. However, the fact that samples were submitted for diagnosis with limited clinical information makes it impossible to further clarify the putative impact of the co-infections with respect to the clinical outcome. The incentive to develop and validate PRV1- and SOV-specific RT-qPCRs was based on reports of swine clinicians about “typical” swIAV-like disease in herds from which no evidence of swIAV infection could be obtained by molecular diagnosis; instead, PRV1 was detected in the majority of such cases. Apart from the contributions of additional bacterial and viral pathogens mentioned as aetiological agents in the PRDC, the findings on PRV1 and SOV occurrences do not refute a putative causal function of these viruses in pig respiratory disease. Studies using a larger number of samples and pig farms are beneficial in determining the prevalence and effect of PRV1 and SOV in Europe. Obtaining cell culture-grown isolates from clinical samples would be necessary for conducting challenge experiments to investigate and describe the potential clinical impact of these viruses according to the Henle-Koch postulates.

We showed continuing diversifying evolution of swIAV with new reassortants between human pandemic H1N1 of 2009 and the avian-derived swine lineage. In addition, we detected a high incidence of PRV1 in pig holdings affected with respiratory disease, both with and without co-infection of swIAV. SOV was detected at lower incidences. We hypothesize that, in addition to swIAV, PRV1 may play a role in respiratory illnesses in pigs in Germany.

Our modified mRT-qPCRs provide robust and updated tools for a rapid and simultaneous detection of three viral respiratory pathogens in pigs needed to conduct sustained monitoring programs in Europe. Further antigenic, in-depth genetic, and biological characterizations of circulating viral strains will require additional virus isolation on selected samples. Given PRV1 and SOV’s potential to induce respiratory disease in pigs, both viruses should be evaluated for differential diagnostic testing in pigs with respiratory disease who are suspected of having swIAV infections.

## Supplementary Material

Supplemental Material

## Data Availability

The datasets used and/or analyzed during the current study are available from the corresponding author on reasonable request.

## List of abbreviations

BLAST: Basic local alignment search tool
Cq: Cycle of quantification
F: Fusion Protein
HA: Hemagglutinin
IAV: Influenza A Virus
M: Matrix gene
NA: Neuraminidase
Nd: not defined
Neg: Negative
NGS: Next generation sequencing
NP: Nucleoprotein
PCV-1: Porcine circovirus type 1
PCV-2: Porcine circovirus type 2
PEDV: Porcine epidemic diarrhea coronavirus
PRV1: porcine respirovirus-1
PRRSV: Porcine reproductive and respiratory syndrome virus
RNA: Ribonucleic acid
RT-qPCR: Semi-quantitative real time RT-PCR
mRT-qPCR: multiplex RT-qPCR
Rxn: Reaction
swIAV: swine influenza A virus
SOV: swine orthopneumovirus
TGEV: Transmissible gastroenteritis (corona)virus
